# Development, Implementation, and Process Evaluation of *Bukhali*: An Intervention from Preconception to Early Childhood

**DOI:** 10.1007/s43477-023-00073-8

**Published:** 2023-03-11

**Authors:** Catherine E. Draper, Nomsa Thwala, Wiedaad Slemming, Stephen J. Lye, Shane A. Norris

**Affiliations:** 1 SAMRC/Wits Developmental Pathways for Health Research Unit, Department of Paediatrics, Faculty of Health Sciences, University of the Witwatersrand, Johannesburg, South Africa; 2 Department of Paediatrics and Child Health, School of Clinical Medicine, Faculty of Health Sciences, University of the Witwatersrand, Johannesburg, South Africa; 3 Lunenfeld-Tanenbaum Research Institute, Sinai Health, Toronto and Departments of Obstetrics and Gynecology, Physiology and Medicine, University of Toronto, Toronto, ON Canada; 4 Global Health Research Unit, School of Human Development and Health, University of Southampton, Southampton, UK

**Keywords:** LMIC, Life-course perspective, Childhood obesity, Intervention development

## Abstract

The Healthy Life Trajectories Initiative, an international consortium developed in partnership with the World Health Organization, is addressing childhood obesity from a life-course perspective. It hypothesises that an integrated complex intervention from preconception, through pregnancy, infancy and early childhood, will reduce childhood adiposity and non-communicable disease risk, and improve child development. As part of the Healthy Life Trajectories Initiative in South Africa, the *Bukhali* randomised controlled trial is being conducted with 18–28-year-old women in Soweto, where young women face numerous challenges to their physical and mental health. The aims of this paper were to describe the intervention development process (including adaptations), intervention components, and process evaluation; and to highlight key lessons learned. Intervention materials have been developed according to the life-course stages: preconception (*Bukhali*), pregnancy (*Bukhali Baby*), infancy (*Bukhali Nana*; birth—2 years), and early childhood (*Bukhali Mntwana*, 2–5 years). The intervention is delivered by community health workers, and includes the provision of health literacy resources, multi-micronutrient supplementation, in-person health screening, services and referral, nutrition risk support, SMS-reminders and telephonic contacts to assist with behaviour change goals. A key adaption is the incorporation of principles of trauma-information care, given the mental health challenges faced by participants. The *Bukhali* process evaluation is focussing on context, implementation and mechanisms of impact, using a mixed methods approach. Although the completion of the trial is still a number of years away, the documentation of the intervention development process and process evaluation of the trial can provide lessons for the development, implementation, and evaluation of such complex life-course trials.

**Supplementary Information:**

The online version contains supplementary material available at 10.1007/s43477-023-00073-8.

## Introduction

Childhood obesity remains a global public health concern, given that adiposity in childhood is a risk factor for poorer health trajectories and the development of non-communicable diseases (NCDs), which contribute significantly to the health burdens in low- and middle-income countries (LMICs) (Wang et al., 2016). The importance of healthy weight and reducing NCD risk amongst women preconception has been highlighted, with evidence showing that pre-pregnancy obesity is predictive of obesity in children (Heslehurst et al., 2019; Woo Baidal et al., 2016). The promotion of healthy behaviours to reduce NCD risk in young women of child-bearing age not only has the potential to improve women’s own health but, can also increase the probability of a healthier pregnancy and baby, should they become pregnant (Fleming et al., 2018; Stephenson et al., 2018). This includes the reduction in obesity risk, both of women and their offspring (Wells et al., 2020). However, despite the attention being given to the preconception period, preconception health remains both understudied and under-resourced in LMICs (Dean et al., 2014; Mason et al., 2014). Added to this are concerns regarding the developmental trajectories and outcomes of young children in LMICs (Britto et al., 2017; McCoy et al., 2022). Furthermore, taking a bio-social life-course perspective (Hanson & Aagaard‐Hansen, 2021), investments in the preconception period should be followed up during pregnancy and infancy and into early childhood. However, there are no published trials of a continuum of interventions through these periods of the life-course.

### Healthy Life Trajectories Initiative

In response to these identified gaps in research, the Healthy Life Trajectories Initiative (HeLTI), an international consortium developed in partnership with the World Health Organization (WHO), is addressing childhood obesity from a life-course perspective. HeLTI hypothesises that an integrated complex intervention, comprising a continuum of care from preconception, through pregnancy, infancy and early childhood will reduce childhood adiposity and the risk for NCDs, and improve child development. This hypothesis is being tested in four randomised controlled trials in Canada (Dennis et al., 2021), China (Wu et al., 2021), India (Kumaran et al., 2021), and South Africa (Norris et al., 2022). HeLTI aims to establish a programme of research to generate evidence that will inform national policy and decision making around preconception health as an intervention opportunity. This evidence focusses on optimising young women’s physical and mental health in order to establish healthier trajectories for themselves and future offspring, and to offset health risks, including obesity.

While there have been substantial efforts to harmonise primary and secondary outcomes and measures, and interventions across the four countries, interventions have been developed to be contextually relevant within each setting. One intervention component that was harmonised across the four countries was the incorporation of training in the Healthy Conversation Skills approach for intervention teams. This approach is intended to maximise health care practitioners’ skills to support and empower patients through the process of behaviour change (Barker et al., 2011; Lawrence et al., 2016).

#### HeLTI South Africa—Bukhali

As part of HeLTI South Africa, the *Bukhali* randomised controlled trial is being conducted in Soweto, a densely populated, predominantly low-income, urban, and multilingual setting in Johannesburg. *Bukhali* means smart or powerful in isiZulu (commonly spoken language in Soweto), with the catchphrase of ‘Living your best life’. Extensive epidemiological research in Soweto has identified young women (18–28 years) as the target population for this trial, given the high proportion (67%) of young women who are overweight or obese during pregnancy (Wrottesley et al., 2020). In addition, the Birth to Thirty cohort study in Soweto showed that if a girl was obese at 5 years old, she had a 42 times greater risk of being an obese adult (Lundeen et al., 2016), and that overweight and obesity increase substantially from childhood (10% at 8 years) to early adulthood (43% at 22 years) amongst females (Nyati et al., 2019). Furthermore, unhealthy diet (Sedibe et al., 2014, 2018; Wrottesley et al., 2017), high sedentary behaviour (Micklesfield et al., 2017; Prioreschi et al., 2017), physical inactivity in late adolescence (Hanson et al., 2019) have been identified as risks for NCDs amongst young women in Soweto. The prevalence of antenatal depression (27%) and anxiety (15%) have been noted as additional risks for young women in this setting (Redinger et al., 2018).

Formative qualitative work conducted for HeLTI highlighted the various socioeconomic challenges faced by young women in Soweto, as well as the environmental, social, and structural constraints (e.g. normalisation of obesity, safety concerns) that make it difficult for them to prioritise their own health when making choices regarding their physical activity and dietary behaviour in Soweto. This qualitative work also drew attention to the complex family dynamics experienced by young women in Soweto, and their need for mental health support (Cohen et al., 2020; Draper et al., 2019; Ware et al., 2019). Additional qualitative work affirmed the reality of these challenges, and highlighted the relative low priority of health, and the “preconception knowledge gap” amongst young women in this setting (Bosire et al., 2021; Draper et al., 2020).

Findings from the *Bukhali* trial pilot data (~ 1600 women aged 18–25 years) have further emphasised how young women in Soweto are exposed to numerous risks in their social and economic environment, including social injustice, gender-based violence and other traumatic events, limited educational and employment opportunities (Ware et al., 2021). Using an adapted social vulnerability index, 27% of the sample were classified as socially vulnerable; this increased to 44% for women with one child, and 64% for women with more than one child (Ware et al., 2021). In this sample of women, 33% of women were classified as food insecure, 20% were at risk of being food insecure, and 44% were overweight/obese (Kehoe et al., 2021). Less than half of the women reported any leisure time physical activity, although 86% of women met physical activity guidelines (based on self-report), but this was typically accumulated through transport- or work-related activity (Prioreschi et al., 2021). Poor quality sleep was reported by a third of the women in this sample, with 19% and 15% were classified as having depression and anxiety respectively, and 24% were at risk for harmful alcohol use (Draper et al., 2022a, 2022b).

## Intervention Development and Implementation

While the trial protocol and intervention strategies have previously been presented, the primary aim of this paper is to more comprehensively describe the following for *Bukhali*: (1) the intervention development process, (2) intervention components, and (3) the process evaluation. Furthermore, drawing on these descriptions, this paper aims to highlight key lessons learned thus far. We utilised information gathered from investigators and documented processes and study data to detail the intervention.

For all the HeLTI countries, the interventions were developed to address childhood obesity (excess adiposity) and informed by the UK Medical Research Council (MRC) Guidelines for Complex Interventions available at the time of intervention development (Craig et al., 2008), theoretically grounded in behaviour change principles, and set out four objectives: (1) optimise health, (2) optimise nutrition, (3) optimise mental health, and (4) promote early childhood development. The original logic model guiding intervention development is presented in Supplementary Fig. 1 (Norris et al., 2022)(reproduced with permission) and an overview of the intervention is presented in Supplementary Fig. 2, which highlights the community health worker (CHW) approach, as well as adaptations to the intervention (discussed below under “Intervention adaptations”), which are highlighted in red.

The *Bukhali* trial and intervention details have been published elsewhere (Draper et al., 2022b; Norris et al., 2022), along with the findings and learnings from the pilot implementation of *Bukhali* (Draper et al., 2020). In brief, the *Bukhali* intervention is delivered individually to participants by ‘Health Helpers’, equivalent to CHWs in South Africa. The application of relevant constructs from the Consolidated Framework for Implementation Research (CFIR) (Damschroder et al., 2009) to the *Bukhali* intervention is provided as Supplementary Table 1. A ‘pragmatic attitude’ has been adopted in the trial design to maximise applicability to usual care settings (Treweek & Zwarenstein, 2009), placing it closer to the pragmatic end of the ‘pragmatic-explanatory continuum’ (Thorpe et al., 2009). This pragmatic inclination can help strengthen the external validity of complex intervention evaluations and provide valuable insights on the feasibility of such interventions; but this external validity needs to be balanced with internal validity (Minary et al., 2019). In the *Bukhali* trial, potential biases that can threaten internal validity (Spieth et al., 2016) are addressed within the trial design (Norris et al., 2022) in order to ultimately strengthen claims about the intervention’s impact. Furthermore, the process evaluation embedded within the trial (described later) help to explicate the mechanisms of this impact (Minary et al., 2019).

The *Bukhali* trial is being implemented to align with ‘real world’ conditions, to maximise the opportunity to inform policy and practice in the South African public health sector, and to facilitate later community-wide implementation and scale-up of intervention strategies within the primary health care system. For this reason, the Health Helpers share similar qualifications and salary levels to CHWs in South Africa, although they are hired specifically for the trial. All Health Helpers are women between the ages of 23–43 years (mean age = 31.9 ± 5.21; median age = 30.93) (Draper et al., 2022b). Intervention delivery includes the provision of health literacy materials with health screening (obesity, anaemia, depression, diabetes, hypertension), feedback and referral, and services and behaviour change through individual sessions in person or telephonically. These services include free HIV and pregnancy testing, and a free curriculum vitae (CV) printing service. Participants also receive multi-micronutrient supplementation as part of the intervention, and these are delivered by a team of drivers who take the supplements to participants’ homes if they cannot be given in-person by Health Helpers.

### Intervention Materials

Intervention materials have been developed according to the life-course stages: preconception (*Bukhali*), pregnancy (*Bukhali Baby*), infancy (birth—2 years), and early childhood (*Bukhali Mntwana*, ‘child’ in isiZulu, 2–5 years). The content of these materials is outlined in Table 1, and selected images are provided in Supplementary Fig. 3. For each stage, content experts have been engaged to provide input on relevant content, and a graphic designer has assisted with the design of materials. Also, young women test groups were involved to promote greater acceptability and value. For the preconception and pregnancy materials, a specialised health curriculum designer was contracted to compile these materials, and for these phases Health Helpers received a manual, and participants received a resource manual.

**Table 1:** Intervention content across the trial phases

Phase	HH resources	Participant resources	Health checks	Dietician sessions	Focus and key topics
Preconception—*Bukhali*	Health Helper’s Manual	*Bukhali* Resource Book	BMI, BP, Hb, free HIV and pregnancy testing	At least 1 session for obese participants	*Focus: Optimising physical and mental health of young women, and build a foundation for early childhood development*
					Body composition and body shape
					Non-communicable diseases
					Healthy eating
					Physical activity and fitness
					Sedentary behaviour and screen time
					Sleep
					HIV
					Contraception
					Sexually transmitted infections
					Emotional health
Pregnancy—*Bukhali Baby*	Bukhali Baby Health Helper’s Manual Breastfeeding support materials	*Bukhali* Baby Resource Book	BMI, BP, Hb, free HIV testing, ultrasound, GDM screening	At least 1 session for overweight and obese participants	*Focus: Optimising physical and mental health of young women, and build a foundation for nurturing care*
					Healthy and safe pregnancy
					Partner and family support
					Care for child development
					MomConnect SMS service
					Healthy eating
					Physical activity
					Preparing for delivery
					Accessing Child Support Grant
					Pregnancy milestones
					Preparing for breastfeeding
Birth—12 months	Counselling cards Care for Child Development resources	Road to Health Book Early years movement guidelines	BMI, BP, Hb, free HIV and pregnancy testing	At least 1 session for obese participants (mother)	*Focus: Promoting nurturing care and establishing a healthy growth and development trajectory for the child*
					Road to Health Book pillars:
					Good nutrition to grow and be healthy
					Love, play and talk for healthy development
					Protection from preventable childhood diseases and injuries
					Healthcare for sick children
					Special care for children who need extra care
					Healthy movement behaviours:
					Physical activity
					Sedentary behaviour
					Screen time
					Sleep
12–24 months	Health Helper’s Manual Road to Health Book Early years movement guidelines	Road to Health Book Early years movement guidelines	BMI, BP, Hb, free HIV and pregnancy testing	At least 1 session for obese participants (mother)	*Focus: Optimising the healthy growth and development of the child*
					Road to Health Book pillars
					Includes feeding guidelines and responsive feeding support
					Healthy movement behaviours
24–60 months—*Bukhali Mntwana*	Health Helper’s Manual Road to Health Book Early years movement guidelines	Road to Health Book Early years movement guidelines Growth chart Diary sheets: screen time, food, sleep, beverages Activity handouts	*Mother:* BMI, BP, Hb, free HIV and pregnancy testing *Child:* Height, weight, developmental milestone checks	At least 1 session for overweight and obese participants (child)	*Focus: Optimising the body composition and development of the child (transition to obesity prevention focus with the child)*
					Child development: fine and gross motor, cognitive, social emotional, numeracy, language and literacy
					Child growth: monitoring height and weight
					Child healthy behaviours: physical activity, sedentary behaviour, screen time, sleep, dietary behaviour
					Maternal well-being: self-awareness, emotional awareness, stress and coping, self-care, values
					Activity @ home: encouraging connection and responsive caregiving
					Continued alignment with Road to Health Book pillars, e.g. immunisations, deworming, feeding, illness

*HH* health helper, *BMI* body mass index, *BP* blood pressure, *Hb* iron, *HIV* human immunodeficiency virus, *SMS* short message service

The infancy intervention materials were informed by the South African child health record. the Road to Health Book (South African National Department of Health, n.d.-a), the WHO/UNICEF ‘Caring for the child’s healthy growth and development’ materials and the Nurturing Care Framework (World Health Organization et al., 2018). Additional components related to support for responsive caregiving and responsive feeding based on the WHO/UNICEF Care for Child Development (World Health Organization & UNICEF, 2012) approach and nationally developed Side-by-Side materials (South African National Department of Health, n.d.-b), were added to the intervention package for children up to the age of two. Health Helpers received a manual, and counselling cards, structured around the Road to Health Book knowledge pillars (nutrition, love, protection, healthcare, and extra care), that guide them through their interactions with mothers and their children.

All the materials have been developed as the trial has progressed, which has enabled a flexible and somewhat iterative approach in terms of adjusting the content and format of the materials for each new stage in response to feedback from the intervention team and participants. Within this flexibility, structure is provided to the sessions in terms of key outcomes to be addressed through the provision of ‘cue cards’ to assist with risk screening, management and referral, and to prompt Health Helpers in their interactions with participants. These follow a ‘traffic light’ system to provide guidance, with green indicating normal range or no risk, orange indicating some or elevated risk, and red indicating high risk and prompting an appropriate referral. An example of these cue cards for blood pressure, iron (Hb), diabetes (HbA1C), body mass index (BMI), and knowledge of human immunodeficiency virus (HIV) status in the preconception stage is provided in Supplementary Fig. 4.

### Control Arm

The control arm components were intended as standard of care ‘plus’ offered through the public health system, assuming that standard of care would be insufficient to engage participants for the full length of the trial (5–7 years if they became pregnant). These components are delivered telephonically by a trained call centre team, with 6-monthly in-person visits to promote contact and reduce attrition. Control participants are also offered free HIV and pregnancy testing, and the same CV printed service as intervention participants. The control component covers non-health specific topics relevant to young women, in order not to contaminate the intervention, but attempts to partially control for the attention given to intervention participants (Norris et al., 2022). Qualitative findings, thus, far from the process evaluation (discussed further below) indicate that this attention is generally well received by control participants.

### Intervention Dosage

Supplementary Figure 5 outlines the dosage of the components of the intervention and control arms (combining birth—60 months into ‘early childhood’). This figure provides details for the: number of health literacy resources provided; allocations of multi-micronutrient supplement (per month, intervention only); in-person sessions, including health checks for intervention (including free HIV and pregnancy testing, CV printing) and services offered for control (only free HIV and pregnancy testing, CV printing); and telephonic contacts. The in-person visits ideally take place every 6 months and these have proved to be crucial to promote adherence and engagement of both intervention and control participants. For intervention participants, these in-person visits are a valuable opportunity to reinforce (and repeat, if necessary) health literacy messages and support provided by Health Helpers.

### Intervention Adaptations

With regard to the various adaptations that have been made to intervention delivery (intervention arm) over the course of the trial, first, the preconception sessions were originally designed to be facilitated in community-based peer group sessions (facilitated by a Health Helper), but these proved not to be feasible in the pilot phase (Draper et al., 2020). Second, delivering in-person sessions in participants’ homes also proved not to be feasible, and individual session shifted to be largely telephonic. This happened in early 2020, which then enabled the intervention team to pivot more quickly during the early stages of the COVID-19 pandemic, when in-person sessions were not possible. The supplementation component of the intervention was allowed to continue during COVID-19 lockdowns. Third, adaptations to the implementation of Health Conversation Skills have had to be made, after finding that the approach could not be delivered as intended in this trial setting, and that contextual adaptations to the approach were necessary. These include simplifying the goal setting planning tool, adaptations for multi-lingual settings and where literacy levels may be low, adapting training to an ongoing trial setting, and incorporating a trauma-informed perspective to health behaviour change (Draper et al., 2022b). Where possible, these adaptations have been incorporated into the delivery of the preconception, pregnancy, and infancy components, and specifically into the intervention materials for the early childhood phase.

Fourthly, in mid-2021, a registered dietician joined the intervention team to provide additional nutritional support for participants who were overweight or obese (especially during pregnancy). These nutrition support sessions were also offered in-person and telephonically, and further details regarding the evaluation of this nutritional support are provided below as part of the process evaluation. Lastly, in 2021, based on the learnings and experience of the intervention team regarding the extent and salience of mental health challenges experienced by participants, along with findings from the process evaluation (Draper et al., 2022b), specific attention was directed towards understanding trauma experienced by participants, and by the Health Helpers as well. This was done to ultimately identify ways in which mental health support could be provided for participants and Health Helpers. Two discussion sessions (~ 2 h in total) about trauma was facilitated with the intervention team (Health Helpers, dietician, and drivers), key issues from this discussion, and steps taken in the trial to further address mental health challenges and trauma are explained below (including recruitment of a social worker on the trial, discussed below under ‘Solutions’), with selected quotes to illustrate these issues in Table 2.

**Table 2:** Intervention team’s perspectives on trauma in the Bukhali trial

*Definition of Trauma*
“I think it’s something that is deep, that is embarrassing you, that you can’t even explain it, something that is deep and eating you inside, even when you explain it to another person, you feel that the person does not know what you are talking about, that is how deep it is, that you can’t even explain it”
“…it’s something that causes you bitter…experience that is emotional that you cannot exactly move on, you keep going back to it, it’s something that you are stuck with”
“…it’s painful and deep…it affects our emotions as well and then it makes you bitter, you can’t even allow things flowing in your mind”
“It’s inside of you, it’s you actually…it’s inside and it’s killing you and it’s not good…it’s broken your mind, when you think about it…when you want to go on, you cannot go on”
“Your character, your behaviour and character, it’s like it’s changing it. I think that is why you have that bitterness, stress, and you are angry”
*Experiences of Trauma*
“As Africans, our background are differ from persons to persons, so some of the things, you find that you think they are normal, you understand, because when you grew up, the man of the house next door was beating the women, you understand, so it’s normal, so, but as time goes by, you find that no, what was happening, it is affecting you as well, as you are growing up”
“I think we are not taught that’s it’s trauma…so as long as you don’t see it as a problem, you deny it, almost, so you go through life just in denial”
“…people think that other people’s traumas are bigger than yours, and people have bigger problems than you, and you are getting eaten alive bit by bit”
“Don’t cry… I think our culture taught us that we must not take trauma very serious, we must take trauma seriously if we say [name] was raped, wow, that’s traumatic, and if they point a gun at you, at least you are still alive…come on, two guns, that is traumatic, that is a trauma, and they say, yeah, we know, but at least she is still alive”
“Sometimes people don’t identify your trauma, and in most cases, society has always taught you, be positive, so when this has happened, be positive”
“For black people I don’t think so, sorry for saying black people, but I think that we come from different backgrounds, like just growing up, it’s not, they don’t teach you with the intention that this is how you deal with trauma, they don’t, you just learn along the way, you have to just move on from it, you can’t stay in that space forever, a lot of people, especially us blacks, a lot of people grew up in situations where you have, you have uncles that were going insane every night, and when you go outside in your underwear and they are chasing you around, so these are things you just grow up with and you leant to get through, because no one has taught you, you actually have to deal with this…you grow up with resentment towards certain people or certain situations because you haven’t dealt with it”
“You are never taught, that okay, this which I am going through, it’s not okay to cope with things in this manner, you are never taught, no one ever goes, you know what, talk it out, yell it out, cry, scream…”
“we always know that we have to be strong for our families…I’m the eldest at home, I know that I always have to be strong, no matter what the situation, I cannot just leave out of the blue, everybody will die…it’s killing me, but there are people that I have to look out for”
“It’s seriously not okay, not to attend to trauma, because I think it slightly kills you inside, because it builds anger”
“you end up lashing out, releasing it on other people”
*Participants’ Trauma*
“I always feel bad…the reason why I feel bad is because you cannot help that woman…I don’t know how to put it, but it doesn’t feel good to know that I cannot go this route with you, I want to, I really want to go with you, but I just cannot, sometimes interventions close me up in this seat that I am in, yes, I want to go over the chair and do something with you, but I can’t, so for me, I wish we can open up an NGO”
“I think for me, I think our participants need people that they can open up to, people who can then, after opening up, to get assistance, because as I always say to you, it’s very painful to open up a wound and not be able to help the person heal the wound”
“I think also, and also, there are people, they are just like use, the participants, they have their own traumas, as a result, we don’t care, we deal about these things every day, I mean grow up, these things happen in life, so just learn to deal with it, so I think also, the health system, it, I don’t know it it’s how they are trained, or it’s how these things are taught, even at school, university levels, that yeah, this is how life should go, so I just don’t know”
“And then now I have to go back to work, wipe my tears, be calm and then listen to other people’s problems and be like, okay yes, okay, healthy eating, okay yes, stress, okay”
*Solutions*
“…I don’t trust you [referring to psychologist] so much, you are not so connected to me, that I must trust you. So the thing, what is happening with participants is that they are not so formal, but when they come here, they feel so comfortable”
“…instead of just talking, because talking sometimes is just not enough. So I think with the counselling thing, they need to know that they [counsellor] have gone through it and they have dealt with it on their own, can they relate to the type of people here in South Africa”
“Not having an outlet, not having a trust environment, it’s very important because it’s one thing for a person to go, here’s a number, I understand completely, Health Helpers are not trained to be counsellors, but you talk to someone for 18 months, you go through them, their life and everything, and then at the end of the day, here is a number, call this number, and then that person does not have 2 s for you, or in the case where you mentioned who had mental health issues, and then the nurse goes no…[You are talking too much.] Exactly”
“I think having a social worker onsite will actually be a good idea because from the little that I have seen coming, like working here is that people tend to trust us more than they would actually trust that referral that we give them… I feel like they would actually come here and have that session done than go anywhere else because Bukhali has built trust with them from when they were recruited…”
“..that’s why we are saying face to face is much better because how do you trust someone, you are just calling, you going to say it’s [name], you don’t know her or who the friends are, who they are going to tell about your problems. I think so many issues around trust, is the other reason why they don’t call”
“…she is crying, so I explain to her, should I refer so that you can speak to someone, and then she says no, I don’t want to talk to other people, I want to talk to you. So I tell her that I am not a counsellor and all of that, and then she says that then we should not continue with it, you see. So I think that if there is a person onsite, I don’t know what else we can do, but a person that is going to be here, when I take her to that person, she will have the trust that okay, if I trust her, then I can trust the other one as well”

Intervention team includes Health Helpers, dietician and drivers

## Participants’ Definition and Experiences of Trauma

Trauma was understood and resonated as a concept but was defined in more experiential and emotional terms, rather than drawing on a clinical or academic definition. The stigma of mental health was acknowledged, and linked by some to culture. They felt that mental health and trauma were not taken seriously in their context, and their experience was that trauma was normalised in their context, although they could see it from a different perspective looking back on the trauma in their upbringing. They explained how trauma was often minimised in their context, e.g. being told that other people have more trauma, and that they should be positive. Many felt the expectation to be “strong” while experiencing trauma, particularly for their family, especially if they were an older or oldest sibling, or they were the head of the household (including for women). Many spoke of not being taught how to deal with trauma, and not knowing how to cope. Unhealthy ways of dealing with trauma mentioned included “blocking off”, denial, hiding how they felt from others, or convincing themselves that “I can handle this”. Healthy ways of dealing mentioned included self-awareness, and talking about their experiences. The consequences of not dealing with it in a healthy way mentioned were anger, lashing out at others, and depression.

Health Helpers and the dietician were all able to easily recount stories of trauma disclosed to them by participants, and some noted how the content of the intervention (e.g. healthy diet) often do not align with the reality of participants’ circumstances. This leaves them feeling sad, “bad”, and helpless because they are aware that they cannot change a participants’ situation. They find it hard to keep a clear line between personal and professional when dealing with participants, and mentioned that participants sometimes trigger their own trauma. Encouragingly, it was expressed that Health Helpers are seen as a trusted source of support, and that participants are more comfortable opening up to them, often making them the first person to hear of traumatic events experienced by participants.

## Participants’ Recommended Solutions

There was agreement that the local referral systems do not work for participants with mental health challenges and/or trauma. The extent to which these referrals can be relied upon was a key issue. Also, phone consultations (often offered by non-governmental organisations) were not perceived to work. They were not convinced of the value of counselling and therapy in their setting, as they mentioned that talking is not enough in their context, since it does not change the situation causing the challenges and/or trauma for participants. However, they agreed that an onsite counsellor is needed for the trial, but they stressed the importance of the counsellor (or psychologist, social worker etc.) needs to be able to relate to participants, and that they should be trustworthy. They believed that *Bukhali* (and by extension, the research centre) was perceived to be trustworthy by participants, and that this trust could be extended to someone offering services onsite, and therefore, as part of *Bukhali*.

### Actions Taken

Following on from these discussions, in March 2022, steps have been taken to provide additional mental health support for Health Helpers in their role. This support will incorporate principals of trauma-informed care, taking into consideration that experiences of trauma can negatively influence behaviour change (Marks et al., 2021). The provision of onsite counselling is also being investigated, bearing in mind that such services need to be feasible, accessible and sustainable. The need for such services has emerged from the experiences of the intervention team who have experienced challenges with referring high-risk participants to over-subscribed and poorly mental health services in the public sector. While there are some free, non-governmental mental health services available for participants in Soweto, these often are delivered telephonically and/or online, and are difficult for young women to access.

## Process Evaluation

Following guidance from the UK MRC on process evaluation (Moore et al., 2015), the *Bukhali* process evaluation is focussing on context, implementation, and mechanisms of impact, using a mixed methods approach. Regarding context, this refers to participants’ family, community, and social environment, as well as their socioeconomic circumstances. Of particular interest are the life circumstances and lived experiences, and how this might influence their experience of the trial, their understanding of health and health behaviours, and their agency regarding their health and health behaviours. Contextual factors that have been identified thus far include logistical challenges of participating in the trial, exposure to trauma, community influences on perceptions and beliefs, social support and the role of families and partners, and other socio-ecological factors influencing their decision making. Ultimately, we hope to understand how these, and other emergent, contextual factors influence how the intervention is received, implemented, and whether it is effective at changing specified outcomes. Of interest as well is how the intervention might influence the context, especially in the long term. For example, does participation in the trial shape the home environment, or the participants’ interpersonal relationships?

In terms of implementation, key questions and considerations include how delivery is achieved and supported, the fidelity of delivery, the dose of delivery, intervention adaptations, and reach of the intervention. Most of these have been discussed above; intervention reach and dosage are captured on the HeLTI data management system, REDCap (Harris et al., 2009, 2019). Regarding fidelity, a protocol for monitoring fidelity has been developed, drawing on the NIH Behaviour Change Consortium Treatment Fidelity Framework. While this framework was found to provide a good foundation for reporting intervention fidelity, some context-specific challenges were identified with applying this framework in a LMIC setting (Soepnel et al., 2022). In particular, two fidelity components, intervention receipt and enactment, were identified as difficult to uphold and monitor in this setting, given that they are centred around the trial participants rather than the individuals delivering the intervention. Intervention receipt refers to the extent to which participants understand and perform cognitive strategies and skills addressed by the intervention during delivery. Intervention enactment refers to the extent to which these strategies skills can be transferred to real-life situations (Soepnel et al., 2022).

With respect to mechanisms of impact, details regarding the pathway to impact are presented in the *Bukhali* logic model (Supplementary Fig. 1) as well as the intervention overview (Supplementary Fig. 2). Specifically, these include the combination of multiple health screenings, referral and management, health literacy, social and behaviour change support, and multi-micronutrient supplementation delivered by Health Helpers. Furthermore, the process evaluation is capturing data on how participants responding to and interacting with the intervention (including Health Helpers, dietician, social worker), how the intervention is working (including unexpected ways in which it is working), and any unexpected consequences of the intervention (and the control arm). Supplementary Table 2 provides details of the completed, ongoing, in progress, and planned process evaluation activities, along with the aim, methods and status of each activity, and process evaluation framework component/s that apply to the activities.

## Key Lessons Learned

The contextualisation of behavioural changes in young women’s lived experiences in Soweto has been a critical learning so far in the *Bukhali* trial. Furthermore, mental health has emerged as a more salient priority than physical health, and the role of trauma is becoming explicitly acknowledged and dealt with in the trial. Intervention delivery is also no longer only focussed on shifting health behaviours, but encompasses the broader role of support that Health Helpers—as CHWs—can offer young women over the sustained period of a multi-stage trial. An additional learning relating to context is that the salience of certain contextual realities may only emerge as a trial progresses, which can impact not only on trial outcomes (e.g. behaviour change), but also on ongoing recruitment and retention of participants. This can also apply to various approaches and frameworks used in implementation and evaluation, which may need contextual adaptations to be used in a way that is most relevant and helpful.

Another key area of learning in the *Bukhali* trial has been the balance of external and internal validity, in terms of maintaining the required flexibility and making necessary adaptations, and a rigorous trial design. The existence of these on a continuum in complex interventions has been well captured by Minary et al (2019), who intersect this continuum with the continuum of process, mechanisms and effects, and the continuum of efficacy studies and implementation research. They acknowledge that this framework need not be rigidly applied, and that a range of methods may be appropriate for the evaluation of complex interventions (Minary et al., 2019). In light of other learnings mentioned above, the added complication of differing contexts lends further weight to the argument against rigid application of such a framework.

## Conclusions

In South Africa, HeLTI provides a unique opportunity to apply a bio-social life-course perspective to intervening with young women and children to promote the establishment of healthy trajectories and offset childhood obesity. Although the completion of the trial is still a number of years away, from an implementation science perspective, the ongoing documentation (and publication) of the intervention development process and process evaluation of the trial can provide short-term learning and benefits in terms of the development, implementation, and evaluation of the *Bukhali* trial. The application of a framework such as the CFIR helps evaluate and understand what works *where* and *why*, which can facilitate learning across contexts (Damschroder et al., 2009); this could have relevance for similar trials in other LMIC settings. Furthermore, applying an implementation science lens to the *Bukhali* trial, currently focussed on evaluating efficacy (phase 2 trial), provides a critical foundation for a future focus on effectiveness (phase 3 trial). When the effectiveness of the *Bukhali* intervention is evaluated within the public health system, implementation will be critical area for investigation.

Although the evaluation of the implementation of the *Bukhali* trial is ongoing, a limitation of this evaluation is that it is unlikely that we are fully capturing the complexity of the intervention, since we are not able to evaluate all components of the intervention implementation at the same level of detail. Related to this, we have selected certain aspects of implementation on which to focus our evaluation efforts, based on the apparent salience of these aspects of implementation. The long-term nature of the trial (and subsequent blinding) makes it difficult to continually compare the results of the outcome evaluation with the evaluation of the implementation of the intervention.

Despite these limitations, lessons regarding the trial complexity are relevant for trialists, especially for those working across multiple stages of the life course. Our findings, thus, far underscore the critical role of process evaluation, and particularly the inclusion of qualitative methods to explore lived experiences of participants, as well as the more nuanced aspects of context, mechanisms of impact and implementation. Furthermore, the pragmatic attitude adopted in this trial, and the use of process evaluation findings throughout the trial process can provide ongoing insights into the ways in which intervention strategies adopting a CHW approach can strengthen health systems. This is highly relevant in LMIC settings where public sector systems are overburdened and fragile, and where CHWs are a more feasible and acceptable approach for optimising the physical and mental health of young women at community level.

## Supplementary Information

Below is the link to the electronic supplementary material.

Supplementary file1 (PDF 34 KB)

Supplementary file2 (PDF 704 KB)

Supplementary file3 (PPTX 2802 KB)

Supplementary file4 (PPTX 486 KB)

Supplementary file5 (PDF 1028 KB)

Supplementary file6 (DOCX 23 KB)

Supplementary file7 (DOCX 22 KB)
