# Supplementary figures and images for: Development, Implementation, and Process Evaluation of Bukhali: An Intervention from Preconception to Early Childhood
Source: Glob Implement Res Appl. 2023 Mar 11;3(1):31–43. doi: 10.1007/s43477-023-00073-8 (PMC10007644; doi:10.1007/s43477-023-00073-8)

# Logic model for programme of intervention to improve physical and mental health in women

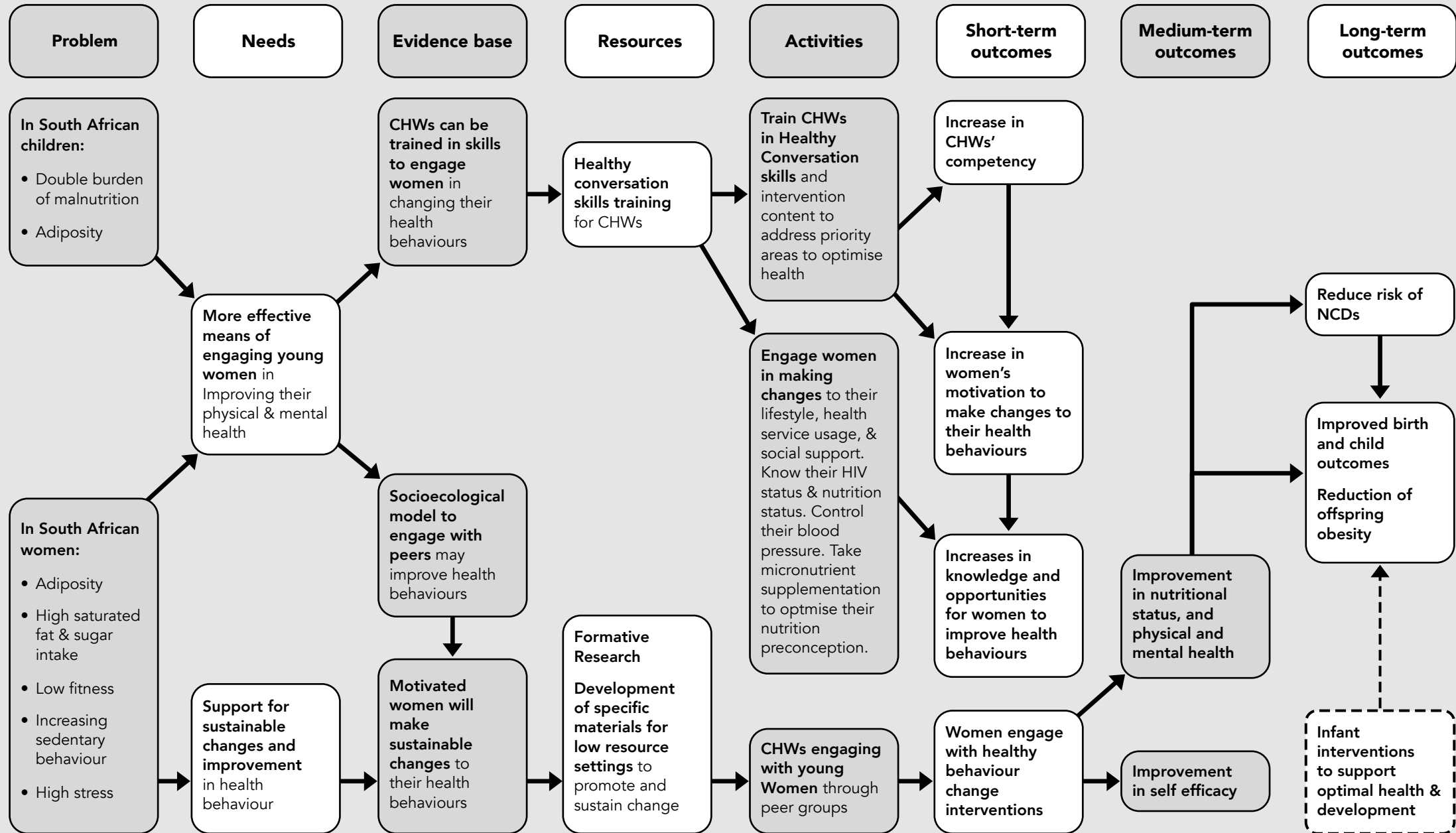

Supplement: Supplementary file 1 — Supplementary file1 (PDF 34 KB) [file 43477_2023_73_MOESM1_ESM.pdf]

## Slide 1
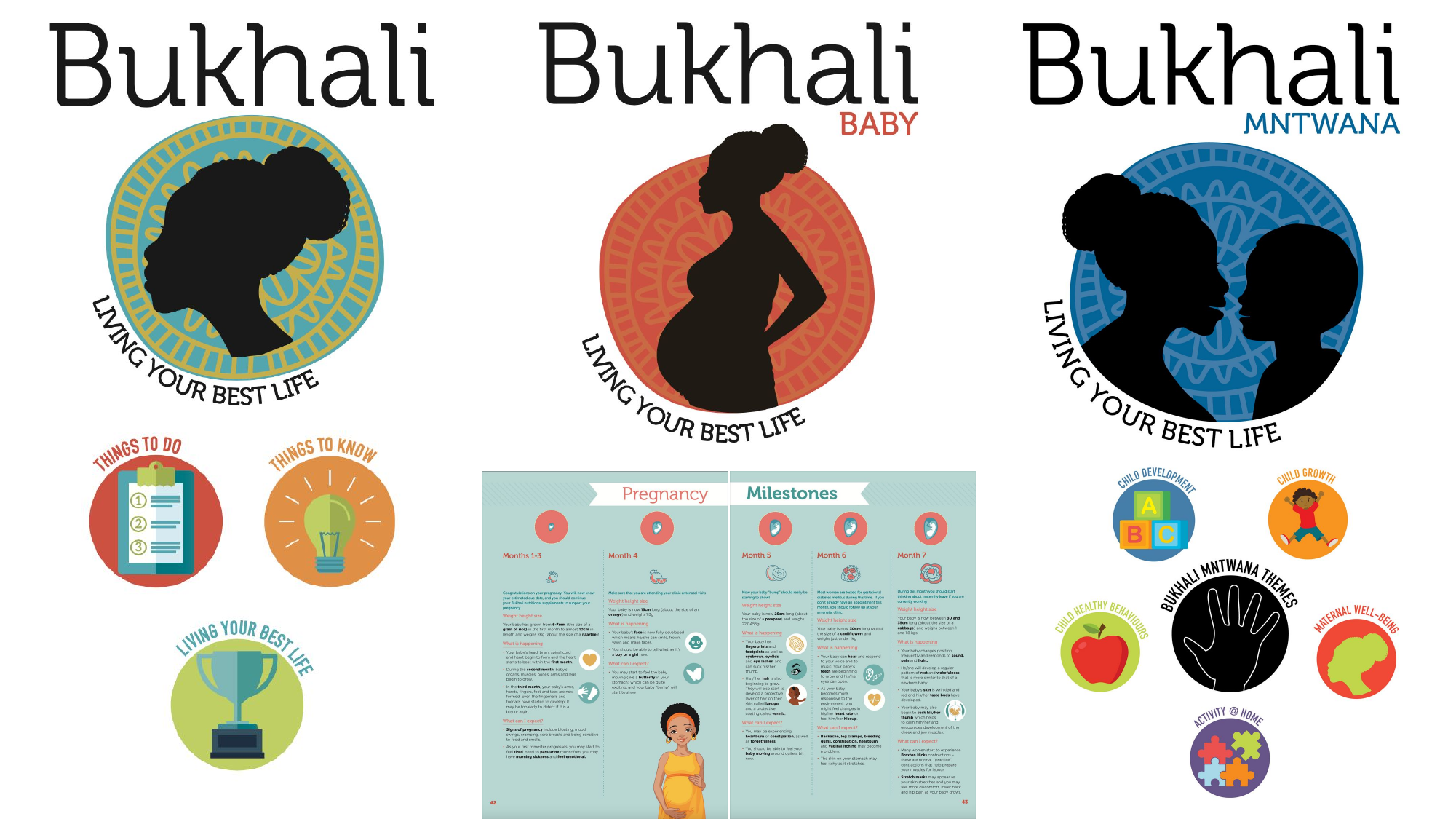

Supplement: Supplementary file 3 — Supplementary file3 (PPTX 2802 KB) [file 43477_2023_73_MOESM3_ESM.pptx]

## Slide 1
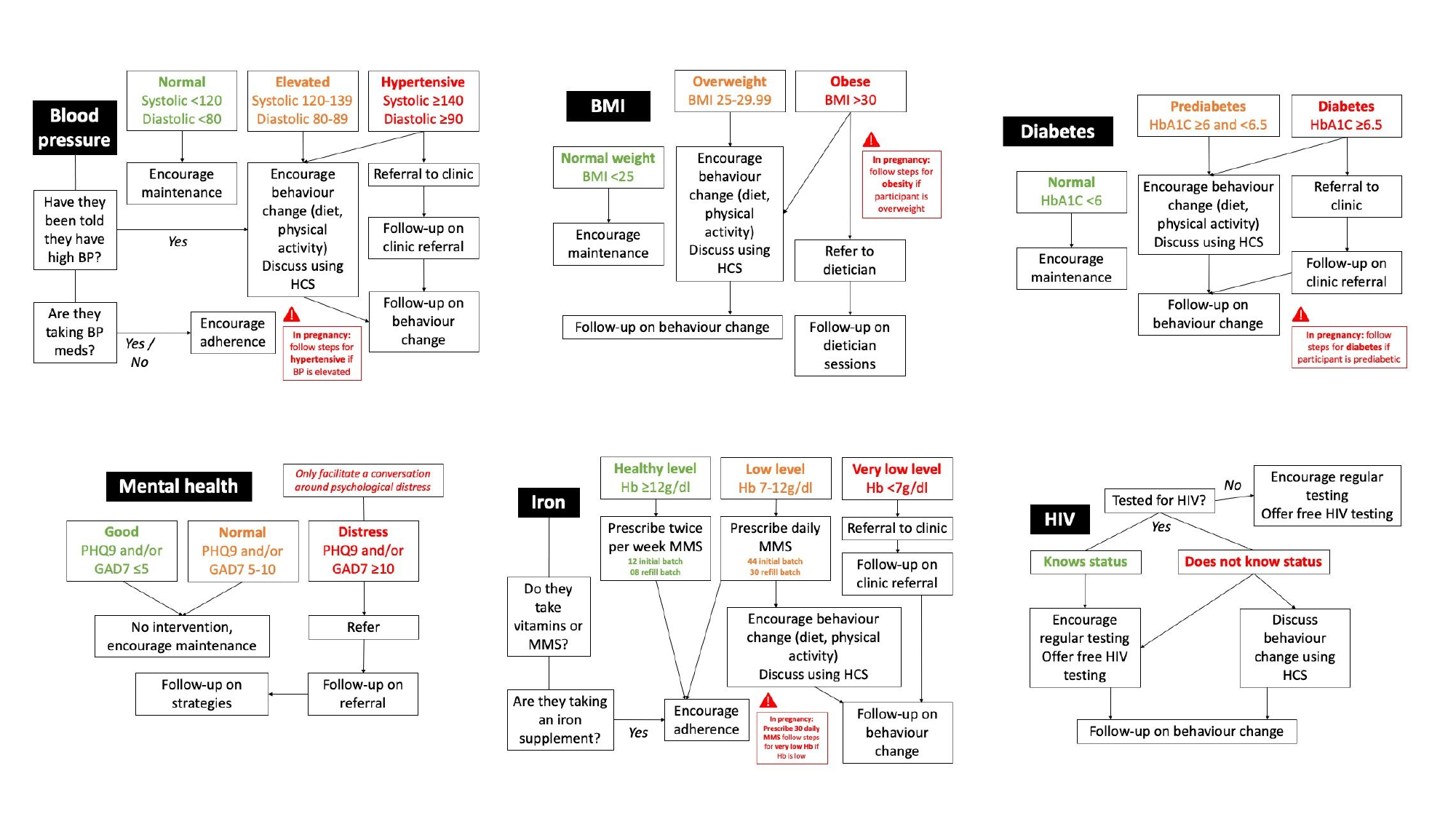

Supplement: Supplementary file 4 — Supplementary file4 (PPTX 486 KB) [file 43477_2023_73_MOESM4_ESM.pptx]
