# Supplementary material for: Development, Implementation, and Process Evaluation of Bukhali: An Intervention from Preconception to Early Childhood
Source: Glob Implement Res Appl. 2023 Mar 11;3(1):31–43. doi: 10.1007/s43477-023-00073-8 (PMC10007644; doi:10.1007/s43477-023-00073-8)
Supplement: Supplementary file 2 — Supplementary file2 (PDF 704 KB) [file 43477_2023_73_MOESM2_ESM.pdf]

Community Health Workers are able to leverage cultural congruence (language, recruited from similar communities); employ interpersonal communication techniques to build trust and rapport; and are trained in Healthy Conversation Skills

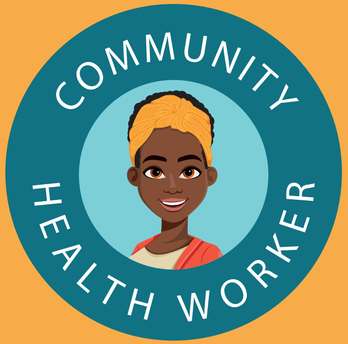

Mental health support for Community Health Workers

Provide risk screening: identify, refer and manage (obesity, anaemia, hypertension, diabetes, depression, anxiety, HIV)

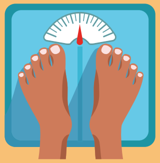

Provide social support: health literacy material, empathy, reinforcement, and access to tools & resources

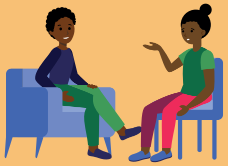

Assist with adopting healthy behaviours: planning & goal setting, enhancing self-efficacy

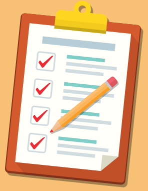

Provide multi-micronutrient supplements: with SMS reminders and support

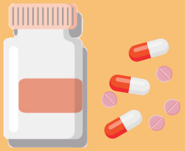

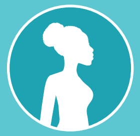

PRECONCEPTION

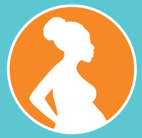

PREGNANCY

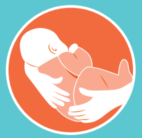

INFANCY

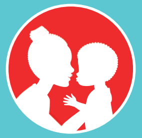

EARLY CHILDHOOD

Mental health support for young women at risk for depression and anxiety

Improved intention for adopting healthy behaviours and managing health risks

Greater adoption of healthy behaviours and managing health risks

Dietician support for overweight/obese women and children

Improved health outcomes
