# Supplementary material for: Development, Implementation, and Process Evaluation of Bukhali: An Intervention from Preconception to Early Childhood
Source: Glob Implement Res Appl. 2023 Mar 11;3(1):31–43. doi: 10.1007/s43477-023-00073-8 (PMC10007644; doi:10.1007/s43477-023-00073-8)
Supplement: Supplementary file 5 — Supplementary file5 (PDF 1028 KB) [file 43477_2023_73_MOESM5_ESM.pdf]

Intervention

	Preconception 18 months	Pregnancy 9 months	Early childhood 60 months
Health literacy resources	3	2	1
Multi-micronutrient supplement	18	5	6
In-person session & health check	3	2	10
Telephonic contacts	9	3	20
Dietician (at least 1 visit for at risk participants)	1	1	1
MINIMUM TOTAL DOSE	33	12	37

Control

	Preconception 18 months	Pregnancy 9 months	Early childhood 60 months
In-person session & services offered	3	1	10
Telephonic contacts	9	3	20
MINIMUM TOTAL DOSE	12	4	30
