# Supplementary material for: Development, Implementation, and Process Evaluation of Bukhali: An Intervention from Preconception to Early Childhood
Source: Glob Implement Res Appl. 2023 Mar 11;3(1):31–43. doi: 10.1007/s43477-023-00073-8 (PMC10007644; doi:10.1007/s43477-023-00073-8)
Supplement: Supplementary file 6 — Supplementary file6 (DOCX 23 KB) [file 43477_2023_73_MOESM6_ESM.docx]

**Supplementary Table 1**

*Application of the Consolidated Framework for Implementation Research to the* Bukhali *Intervention*

| **CFIR Domains and Constructs*** | **CFIR Construct Description*** | **Application to *Bukhali* intervention** |
| --- | --- | --- |
| **Intervention characteristics** | | |
| Evidence strength and quality | Stakeholders’ perceptions of the quality and validity of evidence supporting the belief that the intervention will have desired outcomes. | Extensive evidence on the need to intervene in preconception health (physical and mental) with young women in Soweto. Formative research guided the intervention approach. Stakeholder engagement recognised the importance of the preconception period for intervention and acknowledged very little public health policy, interventions or services exist. |
| Relative advantage | Stakeholders’ perception of the advantage of implementing the intervention versus an alternative solution. | Implementation within public sector health clinics would not be feasible, and formative research indicated a preference for a community health worker-delivered approach, compared to nurse-delivered at a primary healthcare facility. |
| Adaptability | The degree to which an intervention can be adapted, tailored, refined, or reinvented to meet local needs. | The pragmatic nature of the trial allowed for an iterative and flexible approach to intervention development for each intervention stage (preconception, pregnancy, infancy, early childhood), and necessary adaptions of intervention components throughout the stages. |
| Complexity | Perceived difficulty of implementation, reflected by duration, scope, radicalness, disruptiveness, centrality, and intricacy and number of steps required to implement. | The intervention is highly complex, given the 4 stages and multiple components. However, the learning of each components has relevance for implementation strategies within public health and community clinics. |
| Design quality and packaging | Perceived excellence in how the intervention is bundled, presented, and assembled. | Health literacy materials are of a professional standard (expert curriculum developers and inputted by scientists and target group participants). |
| Cost | Costs of the intervention and costs associated with implementing that intervention including investment, supply, and opportunity costs. | Planning for an economic evaluation of the intervention is underway. |
| **Outer setting (external context)** | | |
| Patient needs and resources | The extent to which patient needs, as well as barriers and facilitators to meet those needs are accurately known and prioritized by the organization. | Both extensive formative and ongoing research (qualitative and quantitative) has/is investigating patient needs, barriers and facilitators. This feeds into both adaptions and learning published in academic papers and engagement with stakeholders. |
| External policy and incentives | A broad construct that includes external strategies to spread interventions including policy and regulations (governmental or other central entity), external mandates, recommendations and guidelines, pay-for-performance, collaboratives, and public or benchmark reporting. | The intervention forms part of an initiative (HeLTI), which is supported by the WHO and a global multicounty consortium. HeLTI SA is partly funded through state funding, and intends to inform health policy and practice. |
| **Inner setting (implementing organisation)** | | |
| Structural characteristics | The social architecture, age, maturity, and size of an organization. | The research site has an established reputation for longitudinal health research within Soweto, and many staff have worked on previous projects and reside in/near Soweto. |
| Implementation climate – | The absorptive capacity for change, shared receptivity of involved individuals to an intervention and the extent to which use of that intervention will be rewarded, supported, and expected within their organization. | While both the complex context and nature of the trial and intervention have provided a challenging environment for implementation, creating an optimal implementation climate has been a priority. |
| - Relative priority | Individuals’ shared perception of the importance of the implementation within the organization. | The trial (including other components, in addition to the intervention) is the largest project within the research site, and the importance of supporting implementation is frequently highlighted within the site. |
| - Goals and feedback | The degree to which goals are clearly communicated, acted upon, and fed back to staff and alignment of that feedback with goals. | The goals of the intervention are frequently communicated with the implementation team, and progress is communicated to the leadership. |
| - Learning climate | A climate in which: a) leaders express their own fallibility and need for team members’ assistance and input; b) team members feel that they are essential, valued, and knowledgeable partners in the change process; c) individuals feel psychologically safe to try new methods; and d) there is sufficient time and space for reflective thinking and evaluation. | The leadership aims to provide a positive learning climate for the implementation team, with regular training, and reflective updates and planning. |
| Readiness for implementation – | Tangible and immediate indicators of organizational commitment to its decision to implement an intervention. | Extensive efforts to prepare for implementation of the intervention were made at the start of the trial. |
| - Leadership engagement | Commitment, involvement, and accountability of leaders and managers with the implementation. | Regular engagement occurs with coordinators of various trial components, and specifically the coordinator of the intervention, as well as with community leaders and stakeholders. |
| - Available resources | The level of resources dedicated for implementation and on-going operations including money, training, education, physical space, and time. | Resources are fully provided through grant funding for the trial. |
| - Access to knowledge and information | Ease of access to digestible information and knowledge about the intervention and how to incorporate it into work tasks. | All intervention resources are easily available to all team members and will become fully open access and shareable across the stakeholder network. |
| **Characteristics of individuals (involved in implementation)** | | |
| Knowledge and beliefs | Individuals’ attitudes toward and value placed on the intervention as well as familiarity with facts, truths, and principles related to the intervention. | Training of intervention implementers provides knowledge and skills required to deliver the intervention, and ongoing engagement with the team addresses beliefs of implementers that would influence their implementation of the intervention. |
| Self-efficacy | Individual belief in their own capabilities to execute courses of action to achieve implementation goals. | Training and ongoing engagement intends to promote implementers self-efficacy to deliver the intervention effectively. Supportive leadership and training, and debriefing sessions are in place. |
| Individual stage of change | Characterization of the phase an individual is in, as he or she progresses toward skilled, enthusiastic, and sustained use of the intervention. | Ongoing process evaluation activities helps to assess implementers progress in their ability to successfully deliver the intervention. |
| Other personal attributes | A broad construct to include other personal traits such as tolerance of ambiguity, intellectual ability, motivation, values, competence, capacity, and learning style. | Valued attributes of intervention implementers include empathy and non-judgementalism, as well as the ability to maintain confidentiality, build trust and provide a safe environment for participants. |
| **Implementation process** | | |
| Planning | The degree to which a scheme or method of behavior and tasks for implementing an intervention are developed in advance and the quality of those schemes or methods. | The intervention has been planned, developed and implemented according to the stages mentioned above. This allowed learnings from early stages about implementation (e.g. during preconception) to be incorporated into later stages (e.g. early childhood). |
| Engaging – | Attracting and involving appropriate individuals in the implementation and use of the intervention through a combined strategy of social marketing, education, role modeling, training, and other similar activities. | Intervention implementers are trained in all components of the intervention, and have a team leader who is able to role model effective implementation. |
| - Formally appointed internal implementation leaders | Individuals from within the organization who have been formally appointed with responsibility for implementing an intervention as coordinator, project manager, team leader, or other similar role. | The intervention team leader is employed in a ‘project coordinator’, and maintains regular communication with principal investigators of the trial. |
| Executing | Carrying out or accomplishing the implementation according to plan. | Various mechanisms evaluate the fidelity of intervention implementation. |
| Reflecting and evaluating | Quantitative and qualitative feedback about the progress and quality of implementation accompanied with regular personal and team debriefing about progress and experience. | The process evaluation uses qualitative and quantitative methods to examine the quality of implementation, and regular team debriefs provide further input in this regard. |

*Damschroder, L. J., Aron, D. C., Keith, R. E., Kirsh, S. R., Alexander, J. A., & Lowery, J. C. (2009). Fostering implementation of health services research findings into practice: A consolidated framework for advancing implementation science. *Implementation Science*, *4*(1), 50. <https://doi.org/10.1186/1748-5908-4-50>
