# Supplementary material for: Development, Implementation, and Process Evaluation of Bukhali: An Intervention from Preconception to Early Childhood
Source: Glob Implement Res Appl. 2023 Mar 11;3(1):31–43. doi: 10.1007/s43477-023-00073-8 (PMC10007644; doi:10.1007/s43477-023-00073-8)
Supplement: Supplementary file 7 — Supplementary file7 (DOCX 22 KB) [file 43477_2023_73_MOESM7_ESM.docx]

**Supplementary Table 2**

*Bukhali* Process Evaluation Activities

| **Process evaluation activity** | **Aim** | **Methods** | **Framework component** | **Status** |
| --- | --- | --- | --- | --- |
| Observation of sessions | To observe intervention and control arm sessions to assess fidelity of delivery, and obtain insight into participant-provider interactions. | Structured observation | Implementation  Mechanisms of impact | Ongoing |
| Pilot implementation | To describe the findings and learnings from the pilot implementation of the HeLTI trial in SA, including a description of intervention strategies and adaptations to the trial design. | Qualitative – focus groups  Quantitative – trial monitoring data | Implementation | Completed (published) (Draper et al., 2020) |
| Fidelity | To describe the intervention fidelity protocol for HeLTI SA, based on the NIH Behaviour Change Consortium Fidelity framework. | Document review | Implementation | Completed (in review) (Soepnel, Draper, Mabetha, Dennis, et al., n.d.) |
| Healthy Conversation Skills | To report on the process evaluation of implementing HCS, to identify implementation challenges, and make recommendations HCS adaptations. | Qualitative – focus groups, individual interviews  Quantitative – trial monitoring data | Implementation  Context | Completed (published) (Draper et al., 2022) |
| Supplement use | To understand facilitators and barriers to preconception multi-micronutrient supplement (MMS) adherence, to explore perceptions and beliefs of MMS provision and adherence. | Qualitative – focus groups, individual interviews | Implementation  Context | Completed  (in review) (Silubonde et al., n.d.) |
| Social support during pregnancy | To examine the lived experiences of young women and their receipt of social support during pregnancy, using an interpretive phenomenological approach. | Qualitative – individual interviews | Context | Completed (in review) (Mabetha et al., n.d.) |
| Nutrition | To evaluate the use of a dietary counselling session, including use of the FIGO nutrition checklist (Killeen et al., 2020), to provide context to experiences from dietician and participants’ perspectives, and to determine the prevalence of suboptimal dietary practices (using the FIGO checklist). | Qualitative – individual interviews  Quantitative – FIGO nutrition checklist | Mechanisms of impact | Completed (in review) (Soepnel, Draper, Mabetha, Mogashoa, et al., n.d.) |
| Termination of pregnancy | To understand factors influencing the decision to terminate a pregnancy, applying a socio-ecological perspective. | Qualitative – individual interviews | Context | In progress (manuscript editing) |
| Mobile study | To understand participants’ mobile phone use, and challenges with contacting participants. | Qualitative –interviews  Quantitative – sociodemographic data | Implementation  Context | In progress (manuscript editing) |
| Qualitative longitudinal study | To apply a qualitative longitudinal approach to understand trial participants’ experiences and perceptions, including an interview with participant caregivers (where possible). | Qualitative – individual interviews (multiple over 12 months) | Context  Mechanisms of impact | In progress (analysing data) |
| Partners | To understand the role of participants’ partners (father of the baby) during the pregnancy component of the trial. | Qualitative – individual interviews | Implementation  Context | In progress (data collected) |
| Pregnancy experiences | To explore participants’ perceptions of the impact of the intervention on their pregnancy experiences, compared to a previous pregnancy (prior to the trial). | Qualitative – individual interviews | Mechanisms of impact  Context | In progress (data collected) |
| Screening, management and referral of at-risk participants | To document the screening, management and referral of participants who are identified as at-risk (based on cue cards), and to understand how this process is working from the perspectives of Health Helpers and participants (e.g. barriers to access, challenges of management). | Quantitative – trial monitoring data  Qualitative – individual interviews, focus groups | Implementation  Context | In progress (collecting data) |
| Infancy and early childhood | To understand participants’ perceptions and experiences of caregiving, the role of social support, and trial experiences in infancy and early childhood. | Qualitative – individual interviews, focus groups | Context  Implementation  Mechanisms of impact | Planned |

**References**

Draper, C. E., Mabena, G., Motlhatlhedi, M., Thwala, N., Lawrence, W., Weller, S., Klingberg, S., Ware, L. J., Lye, S. J., & Norris, S. A. (2022). Implementation of Healthy Conversation Skills to support behaviour change in the Bukhali trial in Soweto, South Africa: A process evaluation. *SSM - Mental Health*, *2*, 100132. https://doi.org/10.1016/j.ssmmh.2022.100132

Draper, C. E., Prioreschi, A., Ware, L., Lye, S., & Norris, S. (2020). Pilot implementation of Bukhali: A preconception health trial in South Africa. *SAGE Open Medicine*, *8*, 205031212094054. https://doi.org/10.1177/2050312120940542

Killeen, S. L., Callaghan, S. L., Jacob, C. M., Hanson, M. A., & McAuliffe, F. M. (2020). Examining the use of the FIGO Nutrition Checklist in routine antenatal practice: Multistakeholder feedback to implementation. *International Journal of Gynecology & Obstetrics*, *151*(S1), 51–56. https://doi.org/10.1002/ijgo.13323

Mabetha, K., Soepnel, L., Klingberg, S., Mabena, G., Motlhatlhedi, M., Norris, S. A., & Draper, C. E. (n.d.). *Social Support during pregnancy: A phenomenological exploration of young women’s experiences of support networks on pregnancy care and wellbeing in Soweto, South Africa*.

Silubonde, T. M., Draper, C. E., Baumgartner, J., Ware, L. J., Smuts, C., Lye, S. J., & Norris, S. A. (n.d.). *Micronutrient supplementation among non-pregnant women of reproductive age in Soweto, South Africa: A qualitative study*.

Soepnel, L., Draper, C. E., Mabetha, K., Dennis, C.-L., Prioreschi, A., Lye, S. J., & Norris, S. A. (n.d.). *A protocol for monitoring fidelity of a preconception-life course intervention in a middle-income setting: The Healthy Life Trajectories Initiative (HeLTI), South Africa*.

Soepnel, L., Draper, C. E., Mabetha, K., Mogashoa, L., Mabena, G., Motlhatlhedi, M., McAuliffe, F. M., Killeen, S. L., Jacob, C. M., Hanson, M., & Norris, S. A. (n.d.). *Evaluating implementation of the FIGO nutrition checklist within the Bukhali trial in Soweto, South Africa*.
